# Utility values for symptomatic non-severe hypoglycaemia elicited from persons with and without diabetes in Canada and the United Kingdom

**DOI:** 10.1186/1477-7525-6-73

**Published:** 2008-09-29

**Authors:** Adrian R Levy, Torsten LU Christensen, Jeffrey A Johnson

**Affiliations:** 1 Oxford Outcomes Ltd., Vancouver, BC, Canada; 2 University of British Columbia, Vancouver, BC, Canada; 3 Centre for Health Evaluation and Outcome Sciences, St Paul's Hospital, Vancouver, BC, Canada; 4 Novo Nordisk A/S, Denmark; 5 School of Public Health, University of Alberta, Edmonton, Alberta, Canada

## Abstract

**Objective:**

To elicit societal and patient utilities associated with diabetic symptomatic non-severe hypoglycaemia for three health states: 1) rare (quarterly), 2) intermittent (monthly), 3) and frequent (weekly) hypoglycaemia episodes.

**Methods:**

Using validated health states, time trade-off utilities were elicited from 51 Canadian respondents with diabetes, and 79 respondents in Canada and 75 respondents in the United Kingdom (UK) without diabetes.

**Results and discussion:**

Each hypoglycaemic episode was associated with a reduction in utility and persons with diabetes consistently reported slightly higher utility values than respondents without diabetes. The utility for diabetes without hypoglycaemia ranged from 0.88 to 0.97, the mean utility for rare hypoglycaemic events (quarterly) ranged between 0.85 and 0.94. The utility for the intermittent state (monthly) ranged from 0.77 to 0.90 and from 0.66 to 0.0.83 for the frequent state (weekly). Differences were observed between respondents without diabetes in Canada and the UK. Using a multivariate linear OLS regression, the estimated utilities associated with a single hypoglycaemic event were -0.0033 and -0.0032 for respondents with diabetes and without diabetes, respectively.

**Conclusion:**

Among respondents with and without diabetes, there was a demonstrable utility loss associated with hypoglycaemia. Considering a utility loss of 0.03 as a minimum clinically important difference for persons with diabetes, the evidence from this study indicates that as low as ten symptomatic non-severe hypoglycaemic episodes per year may be of clinical importance and that the importance increases with frequency of episodes. Integrating directly elicited utility values such as those reported here will improve the quality and applicability of economic evaluations of diabetes treatment.

## Background

Hypoglycaemia is a common unintended consequence of insulin that ranges from being bothersome to resulting in coma or even death among persons with diabetes. One group of investigators in the United Kingdom (UK) reported that 73% of insulin users who responded to a mail survey reported at least one hypoglycaemic episode in the past three months [1]. Weekly rates of hypoglycaemic episodes have been estimated at 0.82 and 0.33 for persons with Type 1 and insulin-treated Type 2 diabetes, respectively [2].

While most hypoglycaemic episodes are relatively benign and can be remedied by eating fast-acting carbohydrates, severe episodes – requiring the assistance of others – can result in unconsciousness, seizure, coma and even death. Symptoms of hypoglycaemia can include palpitations, tremor, hunger, and sweating. These can be accompanied by behavioural changes, cognitive impairments, or frank confusion, and can, in severe cases, include seizure, coma, and even death [3].

The American Diabetes Association defines five categories of hypoglycaemia episodes [3]: *severe *(an event requiring assistance of another person to actively administer carbohydrate, glucagons, or other resuscitative actions); *documented symptomatic episodes *(an event during which typical symptoms of hypoglycaemia are accompanied by a measured plasma glucose concentration ≤ 70 mg/dl (3.9 mmol/l)); *asymptomatic episodes *(an event not accompanied by typical symptoms of hypoglycaemia but with a measured plasma glucose concentration ≤ 70 mg/dl (3.9 mmol/l)); *probable symptomatic episodes *(an event during which symptoms of hypoglycaemia are not accompanied by a plasma glucose determination but that was presumably caused by a plasma glucose concentration ≤ 70 mg/dl (3.9 mmol/l)); and *relative episodes *(an event during which the person with diabetes reports any of the typical symptoms of hypoglycaemia, and interprets those as indicative of hypoglycaemia, but with a measured plasma glucose concentration > 70 mg/dl (3.9 mmol/l)).

Hypoglycaemia can have two types of effects on health related quality of life (HRQOL): the short-term consequences of the episode itself and long-term consequences related to behavioural changes and fear of future episodes. The short-term consequences include the unpleasant symptoms related with the actual episodes. The risks of hypoglycaemia are highlighted by dangerous situations that can arise while the patient is hypoglycaemic (e.g. while driving). For example, recently, one of the largest settlements in Canadian legal history was awarded after a person with diabetes suffered a hypoglycaemic episode while driving a car, passed out, and veered into oncoming traffic which led to the death of another driver [4].

The long-term consequences of hypoglycaemia include negative social and emotional sequelae which may make patients reluctant to intensify insulin therapy [5]. The long-term consequences can also include a pattern of fear of hypoglycaemia with negative impact on patients' HRQOL [6]. Qualitative studies on fear of hypoglycaemia relate this fear to loss of control, unpredictability, danger, and interpersonal conflict [7]. This impact can be substantial for both patients and caregivers [1,8,9]. Patients suffering hypoglycaemic episodes are more prone to anxiety and panic attacks and these sequelae increase with the number of episodes [10,11]. In order to avoid hypoglycaemic events, some patients alter insulin treatment intensity and others may engage in behaviours like overeating which are designed to elevate blood glucose levels [12,13]. Fear of hypoglycaemia is consequently commonly observed insulin users [10].

There exist published estimates of directly elicited utilities for symptomatic hypoglycaemia. Currie and colleagues used pooled data from two postal surveys from 1,305 persons with diabetes in Cardiff, United Kingdom (UK) [14]. While benefiting from a large sample, the utilities were elicited from patients. Centralised reimbursement agencies such as the UK's National Institute for Health and Clinical Excellence (NICE) and expert panels typically recommend that economic models incorporate utilities from non-diseased persons [15]. When NICE evaluated the cost-effectiveness of long-acting insulin analogues they also included a factor for fear of hypoglycaemia in the appraisal. However, they also indicated that more research on "fear of hypoglycaemia" was warranted [16].

The objective of the present study was to elicit societal and patient utilities associated with diabetic symptomatic non-severe hypoglycaemia based upon three health states: 1) rare (quarterly), 2) intermittent (monthly), 3) and frequent (weekly) hypoglycaemia episodes. Assessment of severe hypoglycaemic episodes (ADA category 1) was excluded from the study because the clinical symptoms and the consequences of severe events (seizure, coma, and even death) are qualitatively different from less severe events and would be better assessed in a separate study. Asymptomatic episodes (ADA category 3) were also excluded since it would be difficult to describe asymptomatic episodes.

## Methods

Trained interviewers directly elicited utilities using the time trade-off (TTO) method. This method elicits respondents' trade-offs between duration and quality of life. The TTO method is anchored in the fundamental axioms of utility theory, where decision making should involve elements of uncertainty [17]. Respondents are asked what proportion of the remainder of life (e.g., 30 years) they would sacrifice in return for being relieved of the health state and going to perfect health. The higher the proportion the respondent is willing to forgo in exchange for perfect health, the greater the burden attributed to the health state [18].

### Respondents

As there is no consensus whether persons naïve to the disease under study or patients suffering from the disease provide more appropriate data on utilities [15,19], information was collected from both groups. However, given that reimbursement agencies such as NICE recommend utility data obtained from lay-persons [20], we aimed to recruite more persons without diabetes: 75 persons without diabetes in both the UK and in Canada to represent the general population and 50 persons with diabetes in Canada to represent the patients' perspectives. Respondents without diabetes had to be at least 18 years old (17 in the UK) and were recruited through newspaper advertisements. Canadian respondents without diabetes were given $60CDN in supermarket gift certificates and UK respondents without diabetes were paid £20. Excluded were persons who were unable to communicate in English.

Respondents with diabetes had to be: diagnosed with type 1 or type 2 diabetes; 18 to 90 years old; insulin users for at least one year prior to recruitment; and not suffering from visual impairment. Respondents with diabetes were recruited at a diabetes clinic in Vancouver, Canada. These respondents were not remunerated.

The study received ethical approval in the UK and in Canada and complied with the tenets of the Declaration of Helsinki.

### Standardised Descriptions of Health States

The derivation of health states was split into developmental and validation phases. In the developmental phase, four clinical and research experts (three in Canada and one in the UK) were asked to 1) describe the base-case health state for a "typical" person with diabetes on insulin therapy without hypoglycaemia, taking into account five domains from the EQ-5D (mobility, self-care, usually activities, pain/discomfort, and anxiety/depression) and 2) describe the three hypoglycaemia-related health states (rare, intermittent and frequent) using the HFS^© ^[13,14] and relevant clinical literature [12]. The HRQOL dimension in the three hypoglycaemia-related health states were based on the hypoglycaemic fear survey (HFS^©^) [6], a validated instrument which assessed the behavioural and emotional impact of hypoglycaemia [21]. The health states consisted of three sections. The first section described the short-term consequences related with a symptomatic non-severe hypoglycaemic episode ("*If my blood sugar becomes low I feel shaky, dizzy and sweaty. I also get hungry, feel sick and get headaches*"). The second section described the episode frequency (e.g. *"This happens to me about once a month"*). The last section in each health state described the long-term consequences and precautionary measure (e.g. "*I occasionally limit my travelling, driving or social engagements and I sometimes limit the amount of exercise I do*").

In the validation stage, the preliminary health state descriptions were circulated to the experts. Five pilot interviews and cognitive debriefings were conducted in each country to adjust for inconsistencies and language. The consensus health state descriptions are shown in Table 1.

**Table 1:** Standardized descriptions used to characterize health states for hypoglycaemia.

Health State	Description
Baseline Diabetes	I have an illness called diabetes which means that my body cannot keep my blood sugar at a constant level To control this I have to keep to a special diet and be careful about eating regularly I have to inject myself with medication (insulin) on a daily basis, this was difficult at first but I am now used to this and it is not painful I also need to check my blood sugar from time to time, this can hurt a bit I don't have any problems looking after myself I do have to plan my time more than I used to so I know when I am going to eat and exercise I am anxious about the future because I know that diabetes makes me more at risk for other illnesses such as heart disease

Rare (quarterly)	If my blood sugar becomes low I may feel shaky, dizzy and sweaty I may also get hungry, feel sick and get a headache. This only happens to me 3 to 4 times per year To treat this I may eat more to keep my blood sugar high enough. I rarely worry about being criticized for having my low blood sugar level interfere with important tasks and about becoming too emotional. I rarely worry about being able to recognize and control my low blood sugar or acting in an embarrassing way in public, such as appearing drunk and acting aggressively. I am aware of my low blood sugar when I am travelling, driving or in social engagements. I rarely worry about the symptoms of low blood sugar affecting my driving skills or causing injury to myself or others. I sometimes limit the amount of exercise I do. I rarely take measures to ensure I have others with me or checking on me during the day or night.

Intermittent (monthly)	If my blood sugar becomes low I feel shaky, dizzy and sweaty I also get hungry, feel sick and get headaches. This happens to me about once a month. To avoid this I have to keep to my diet and I sometimes measure my blood sugar to check I am OK. I often eat more to keep my blood sugar high in social situations or when doing important tasks. I sometimes worry about being criticized for having my low blood sugar level interfere with important tasks and sometimes worry about becoming too emotional. I often worry about the symptoms of low blood sugar affecting my driving skills and sometimes worry about causing injury to myself or others. I occasionally limit my travelling, driving or social engagements and I sometimes limit the amount of exercise I do. I sometimes take measures to ensure I have others with me or checking on me during the day or night.

Frequent (weekly)	If my blood sugar becomes low I feel shaky, dizzy and sweaty I also get hungry, feel sick and get headaches This happens to me on a weekly basis To avoid this I am very careful about keeping to my diet and I often measure my blood sugar to check I am OK. I often keep my blood sugar high in social situations or when doing important tasks. I always worry about having my low blood sugar level interfere with important tasks and being criticized for it. I always worry about being criticized for having my low blood sugar level interfere with important tasks and sometimes worry about becoming too emotional. If often worry that if my blood sugar gets very low I may become unconscious I often worry about the symptoms of low blood sugar affecting my driving skills or causing injury to myself or others. I always limit my travelling, driving or social engagements and I sometimes limit the amount of exercise I do. I sometimes avoid sex because of the risk of low blood sugar I often take measures to ensure I have others with me or checking on me during the day or night.

### Interview process

Data were collected through one-on-one interviews. The data collection was standardized by training all interviewers and using standardized scripts. Each interview consisted of the respondent reading a short non-technical description of diabetes and hypoglycaemia, a review of the health states (without the name) and a description of the TTO process and a description of the probability board prop. The interviewers presented the health states in different order and respondents were asked to order them from least to most severe to ensure they understood the task.

The utilities associated with each health state were estimated using the TTO technique in which respondents choose repeatedly between 1) remaining in the health state without improvement for 30 years or 2) trading a number of remaining years of life in full health for receipt of a hypothetical treatment that would restore the person to full health. The process incorporated a "ping-pong" approach with probabilities traded back and forth between higher and lower values that iteratively narrowed to the point of indifference [22].

Age, sex, and age at last year of formal education were documented as demographic description for all respondents. For the population with diabetes, descriptive clinical data were also collected.

### Data analysis

For each health state, the TTO utility weight was calculated by dividing the number of years the respondent would live in perfect health by 30 years (for example, if 26 years in perfect health was valued equally with 30 years in the 'diabetes health state', the elicited utility was 0.867). Mean TTO utilities and the corresponding 95% confidence intervals (95% CI) were calculated.

Statistical testing of differences in utility controlled for other confounding factors was conducted using a multivariate linear regression model (i.e., Ordinary Least Squares (OLS)) and a Fractional Logit Generalized Linear Model (FLogit GLM) [23]. A random effects model, which took into account within-respondent variation, was also considered. However, as the within-respondent variation was found negligible only the results from OLS and FLogit regressions are presented.

An OLS model provides regressions coefficients (marginal utilities) which can be easily interpreted and used as input in health economic models. However, the OLS model does not restrict the predicted utility to have an upper limit of 1 (which is a definitional upper boundary). Therefore FLogit models were also estimated [24]

The regression models were used to estimate the disutility associated with a single hypoglycaemic event, after controlling for effects of age (in years), sex, and a dichotomous variable representing education beyond 16 years of age. For respondents without diabetes, we included a variable for country (UK or Canada) and for respondents with diabetes, we included the diabetes type (type 1 or type 2), HbA1c-%, and insulin dosing (units per day).

Two-sided tests with alpha of 0.05 were used. To evaluate the magnitude of the difference in utilities we took 0.03 as a conventional benchmark for clinically important differences on measures of health utility for people with diabetes [25,26]. STATA version 10.0 (StataCorp LP, College Station, Texas) was used for all analyses.

## Results

The final sample consisted of 50 respondents with diabetes in Canada, 79 respondents without diabetes in Canada, and 75 respondents without diabetes in the UK. Respondents with diabetes tended to be older and slightly more than one-half were women (Table 2). Almost 80% of respondents with diabetes were Caucasian and about half were afflicted with at least one other co-morbid condition.

**Table 2:** Demographic characteristics of diabetic respondents in Canada and non-diabetic respondents in Canada and the United Kingdom from whom time trade-off utilities for hypoglycaemia were elicited.

*Characteristic*	*Canadians with diabetes* *(N = 51)*	*Canadians without diabetes* *(N = 78)*	*UK respondents without diabetes* *(N = 75)*
Men (%)	24 (47)	39 (50)	31 (41)
Age, y-mean (SD)	55 (12)	47 (16)	46 (15)
Fully employed (%)	25 (49)	33 (42)	31 (43)
Education after age 16 y (%)	50 (98*)	70 (100*)	63 (85*)
Married or common-law (%)	28 (55)		
Mean daily number of injections (SD)	3.8 (0.9)		
Type 1 diabetes (%)	25 (49)		
Total insulin dose per day (insulin units) (SD)	63 (44)		
Last HbA1c-% (SD)	7.9 (1.3)		
Race:			
White (%)	41 (80)		
Asian (%)	10 (20)		
Number of co-morbidities			
0 (%)	26 (51)		
1 (%)	21 (41)		
2 (%)	4 (8)		

Co-morbidities:			
Cancer (%)	2 (4)		
Cardiovascular (%)	7 (14)		
Depression (%)	6 (12)		
Respiratory (%)	4 (8)		
Renal (%)	5 (10)		
Other*** (%)	5 (10)		

* % of valid responses

** Other conditions included Hypertension, thyroid condition, pancreatitis, retinopathy

For diabetes without hypoglycaemia the mean utilities ranged from 0.88 to 0.97, for rare hypoglycaemic events (quarterly) they ranged between 0.85 and 0.94, for the intermittent state (monthly) they ranged from 0.77 to 0.90, and for the frequent state (weekly) they ranged from 0.66 to 0.83 (Table 3).

**Table 3:** Mean time trade-off utilities (95% confidence intervals) for diabetes alone and four hypoglycaemia health states elicited from diabetic respondents in Canada and non-diabetic respondents in Canada and the United Kingdom

Health state	Canadians with diabetes (N = 51)	Canadians without diabetes (N = 78)	UK respondents without diabetes (N = 75)
Diabetes	0.92 [0.67–1.00]	0.88 [0.62–1.00]	0.97 [0.85–1.00]
Rare hypoglycaemia	0.91 [0.69–1.00]	0.85 [0.55–1.00]	0.94 [0.80–1.00]
Intermittent hypoglycaemia	0.87 [0.63–1.00]	0.77 [0.38–1.00]	0.90 [0.74–1.00]
Frequent hypoglycaemia	0.75 [0.42–1.00]	0.66 [0.22–1.00]	0.83 [0.64–1.00]

* square bracket indicates that the interval was truncated at 1.00

For all three groups, the same rank ordering of health states was observed: lower utilities were observed with more frequent hypoglycaemic episodes. Within each health state, respondents without diabetes in the UK consistently reported the highest mean utility, respondents without diabetes in Canada reported the lowest mean utility, and Canadian respondents with diabetes were intermediate.

Among respondents without diabetes, the OLS regression indicated that four independent variables were associated with utilities for fear of hypoglycaemia (Table 4). The regression R^2 ^was 0.29 (which can be interpreted as this set of predictors accounting 29% of the variation in the observed data). Each hypoglycemic episode was associated with a statistically significant reduction in utility of 0.0032. Men reported utilities 0.0343 higher than women (p < 0.005). Among respondents with diabetes, the OLS regression indicated five of the independent variables were associated with utilities for fear of hypoglycaemia. The regression R^2 ^was 0.28. Each hypoglycemic event was associated with a significant reduction in utility of 0.0033. Respondents with greater educations reported 0.0562 higher utility. Respondents with type 2 diabetes reported 0.0629 higher utility than respondents with type 1 with diabetes. Each insulin unit per day was associated with 0.0008 lower utility.

**Table 4:** Multivariate regressions of utility for respondents with and without diabetes.

	OLS regression		FLogit regression	
	Coefficient	Standard Error	Coefficient	Standard Error
Respondents without diabetes (from Canada and UK)				
Hypo Frequency	-0.0032^†^	0.0002	-0.0235^†^	0.0021
Age	> -0.0000	0.0004	-0.0004	0.0031
Sex (1 = man)	0.0343^†^	0.0119	0.2826^†^	0.0988
Education (1 = long)	0.0110	0.0119	0.0807	0.0964
Country (1 = UK)	0.1156^†^	0.0118	0.9835^†^	0.0902
Constant	0.8578^†^	0.0219	1.9151^†^	0.1714
Respondents with diabetes (from Canada only)				
Hypo Frequency	-0.0033^†^	0.0004	-0.0247^†^	0.0034
Age	-0.0004	0.0008	-0.0026	0.0069
Sex (1 = man)	0.0352	0.0189	0.3023	0.1614
Education (1 = long)	0.0562^‡^	0.0225	0.5007^‡^	0.2098
HbA1c	-1.0728	0.7435	-9.8007	5.1640
Diabetes type 2	0.0629^†^	0.0219	0.5628^†^	0.2169
Insulin dose per day	-0.0008^†^	0.0002	-0.006^†^	0.0016
Constant	0.9857^†^	0.0811	2.956 ^†^	0.5462

† P < 0.01. ‡ P < 0.05

OLS = Ordinary Least Squares

The two Flogit models showed the same four independent variables as the OLS models (Table 4). However, the OLS and FLogit coefficients cannot be directly compared and the latter, while holding the theoretical advantage property of bounding utility at 1, is less straightforward to interpret. Instead of the slope coefficients being the rate of change in utility (the dependent variable) as 'x' (the independent variables) changes, as in the OLS regression, the FLogit slope coefficient is interpreted as the rate of change in the "log odds" as 'x' changes. This interpretation is not intuitive as is shown in the following example which compares the marginal utility estimates from OLS and FLogit. Imagine a 60-year old woman, with no education after her 16^th ^birthday, type 1 diabetes requiring 50 units of insulin per day and an HbA1c-% of 8.0. The utility for this woman with 20 symptomatic hypoglycaemic events per years is estimated to 0.7693. For the same woman 21 hypoglycaemic events yields a utility estimate of 0.7649. Thus, the marginal utility of one hypoglycaemic event is 0.0044. However, due to the non-linear nature of FLogit the marginal utility going from 50 to 51 symptomatic hypoglycaemic events per year is estimated to 0.0059.

## Conclusion

While the utility reduction for rare quarterly hypoglycaemic episodes was very small we observed that respondents reported increasingly large utility reductions from more frequent hypoglycaemic episodes. We also observed differences between countries and respondents groups (with or without diabetes). Increasing insulin dose was associated with lower utility, perhaps because this was as an indicator for disease severity or a higher bodyweight requiring more insulin.

Of the investigators who have published utilities specifically for hypoglycaemia, six have been published in peer-reviewed journals [1,14,16,27-29], and most of these used an indirect measure (the EQ-5D) to derive utilities. While the EQ-5D allows decision-makers to compare across disease states, it was not designed to measure specific health problems associated with hypoglycaemia. The development of time-trade-off health states based on the HFS^© ^directly address diabetic hypoglycaemia.

The estimated mean utility reductions associated with a single non-severe hypoglycaemic episode in this study are of the same order of magnitude than previous studies respectively showing a utility reduction of 0.0052 per symptomatic episode and 0.0035 per symptomatic episodes (annualised from a quarterly utility value of 0.0141) [14,16]. Using the utility value of 0.03 which has been suggested as a benchmark for minimum clinically important differences in utility for persons with diabetes, the evidence from this study indicates that as low as ten symptomatic non-severe hypoglycaemic episodes per year are of clinical importance and that this increases with frequency of episodes [25,26].

Canadian respondents with diabetes reported higher mean utilities than non-diabetic respondents. Higher utility may be due to response shift, to patient adaptation or to the general public's exaggerated fear of the morbidity and disability associated with diabetes [21,22]. Respondents without diabetes received a remuneration fee for their participation in this study. While it is possible that the payments led to the participants responding in socially desirable fashion, that possibility must be weighed against the advantage that remuneration helps to avoid potential selection bias which might have resulted from the omission of those who declined to participate because they put a greater value on their time. Furthermore, offering lay persons compensation for their time is a common practice in this type of research and may therefore improve the comparisons with values reported in other studies.

This study was subject to several limitations. First, TTOs were developed for trading with certainty years of life to avoid a chronic health state [12,24]. The theoretical underpinnings are not in place for the current usage in which TTOs were applied to health states of limited duration. However, the method has been applied to other acute conditions like pertussis and vaccination [25]. One advantage is that TTOs are accepted by reimbursement agencies such as NICE. Second, recruitment was undertaken in only one city in each country, and respondents may not have been broadly representative. However, this study is the largest yet undertaken to directly estimate utilities for hypoglycaemia. Third, the OLS regressions results are only valid within certain pre-set boundaries which is relevant because the OLS model predicting utility values outside 0.0 and 1.0.

Given that a growing number of individuals will require insulin in the future, it can be expected that the burden of hypoglycaemia will also rise [30]. The values herein will aid in informed decision-making by allowing reimbursement authorities to quantify how persons with and without diabetes value health states related to hypoglycaemia. The boundaries in this field of inquiry could be profitably expanded by future studies testing for differences in utility among lay persons in other countries, as well as between persons with the two types of diabetes and different durations since onset.

## Competing interests

The study was undertaken by Oxford Outcomes Ltd., a consultancy specialising in contract research for a wide range of clients in the life sciences industry, including public sector organisations as well as pharmaceutical and other private companies. Funding was provided by Novo Nordisk A/S Denmark. Torsten Christensen is an employee of Novo Nordisk A/S Denmark. Jeffrey A. Johnson received consultancy fees for this project.

## Authors' contributions

ARL contributed to the conception and design, acquisition and interpretation of the data, was primarily responsible for drafting the manuscript, and revising the article critically for important intellectual content. TC contributed to design of the study, analysis of the data, interpretation of the results, and drafting the manuscript. JAJ contributed to the conception and design, interpretation of the data, and revising the article critically for important intellectual content. All authors read and approved the final manuscript.
